# Identification of Drugs Inducing Phospholipidosis by Novel in vitro Data

**DOI:** 10.1002/cmdc.201200306

**Published:** 2012-09-03

**Authors:** Markus Muehlbacher, Philipp Tripal, Florian Roas, Johannes Kornhuber

**Affiliations:** a Department for Psychiatry and Psychotherapy, University Hospital, Friedrich Alexander University Erlangen NurembergSchwabachanlage 6, 91054 Erlangen (Germany) E-mail: johannes.kornhuber@uk-erlangen.de; b Computer Chemistry Center, Friedrich Alexander University Erlangen NurembergNägelsbachstr. 25, 91052 Erlangen (Germany)

**Keywords:** cationic amphiphilic drugs, lysosomal storage disorders, phospholipidosis, phospholipids, toxicology

## Abstract

Drug-induced phospholipidosis (PLD) is a lysosomal storage disorder characterized by the accumulation of phospholipids within the lysosome. This adverse drug effect can occur in various tissues and is suspected to impact cellular viability. Therefore, it is important to test chemical compounds for their potential to induce PLD during the drug design process. PLD has been reported to be a side effect of many commonly used drugs, especially those with cationic amphiphilic properties. To predict drug-induced PLD in silico, we established a high-throughput cell-culture-based method to quantitatively determine the induction of PLD by chemical compounds. Using this assay, we tested 297 drug-like compounds at two different concentrations (2.5 μm and 5.0 μm). We were able to identify 28 previously unknown PLD-inducing agents. Furthermore, our experimental results enabled the development of a binary classification model to predict PLD-inducing agents based on their molecular properties. This random forest prediction system yields a bootstrapped validated accuracy of 86 %. PLD-inducing agents overlap with those that target similar biological processes; a high degree of concordance with PLD-inducing agents was identified for cationic amphiphilic compounds, small molecules that inhibit acid sphingomyelinase, compounds that cross the blood–brain barrier, and compounds that violate Lipinski’s rule of five. Furthermore, we were able to show that PLD-inducing compounds applied in combination additively induce PLD.

## Introduction

Phospholipidosis (PLD) is characterized by an excessive accumulation of phospholipids, which occurs mainly in lysosomes. This effect is often observed upon administration of certain drugs, which is referred to as drug-induced PLD.[1] The accumulation of phospholipids within the lysosomes is a consequence of modified phospholipid metabolism, which is characterized by the occurrence of intracellular multilammelar bodies,[2] vacuolated lymphocytes, and foamy macrophages.[3] Other consequences and effects that are most likely caused by or associated with PLD are still under discussion.[4] Dysregulation of the phospholipid metabolism can be induced by drug application.[5] For example, dibucaine-induced PLD in simian kidney cells resulted in disruption of the actin cytoskeleton, induction of autophagy, reduced proliferation, and an increased rate of cell death.[6] Similar observations were reported for the well-known PLD-inducing drugs amiodarone (**12**) and imipramine (**87**).[4] Whether PLD also leads to an increased rate of apoptosis is still a matter of debate.[4] The cellular consequences of a dysregulated lysosomal phospholipid metabolism underline the relevance of drug-induced PLD.[5], [7] Thus, detailed knowledge about PLD during the drug design process would reduce the risk of adverse effects.[8]

Lüllmann et al. reported in 1978 that many cationic amphiphilic drugs (CADs) induce PLD,[9] and this observation was revisited by Kodavanti et al.[10] Moreover, it has been proposed that CADs are well suited to form synergistic interactions with lipid bilayers.[11] CADs are able to interact with both the carbon core region and the polar head groups of lipid membranes.[12] Thus, CADs easily interact with biological membranes.[13] CADs are present in almost all categories of commonly used drugs, so there is a great deal of interest in this group of compounds.[12] Tricyclic antidepressants, such as amitriptyline (**14**) or desipramine (**56**), are prime examples of CADs.[14] Remarkably, a connection between antidepressant drugs and PLD induction was proposed by Xia et al.[14]

There is still discussion about the molecular mechanism that causes drug-induced PLD.[15] Decreased activity or inhibition of lysosomal phospholipases might cause PLD, as this would result in a lower rate of lysosomal phospholipid degradation.[15], [16] The formation of complexes between phospholipids and PLD-inducing drugs could be another possible mechanism for drug-induced PLD.[9], [17] PLD may also be induced by chemical compounds via the functional inhibition of lysosomal phospholipases. Inhibition of lysosomal acid sphingomyelinase (ASM) occurs in a similar manner.[18]

It is assumed that PLD is linked to clinically relevant consequences.[19] The most frequently reported clinical complication related to drug-induced PLD is liver toxicity.[20] In particular, the well-known PLD-inducer amiodarone (**12**) has been reported to cause toxicity or general damage to the liver.[19], [20b], [21] The application of PLD-inducing drugs, such as fluoxetine (**79**),[20c] has also been frequently reported to cause other clinical effects, such as lung toxicity.[22] In general, lysosomal storage diseases are often related to altered phospholipids levels. Accumulation of sphingomyeline due to a mutation in the gene encoding sphingomyelinase is responsible for Niemann-Pick’s disease. Accumulation of ceramide, the metabolite of sphingomyeline, characterizes Farber’s disease. In addition to these pathologically and clinically relevant issues, recent investigations have shown that certain drugs lead to the accumulation of phospholipids within the central nervous system (CNS).[20a], [23] However, the consequences of dysregulation of phospholipid metabolism for the CNS remain unclear, and a direct correlation between lysosomal phospholipid levels and clinical parameters is still missing. We hope that detailed clinical studies will provide valuable insights into the clinical consequences of drug-induced PLD. Nevertheless, mild drug-induced PLD might also lack significant clinical relevance.

As drug-induced PLD is caused upon drug application, it is per definition a side effect of applied medication. In many clinical situations, medication is vitally important, so adverse side effects are ignored or accepted. However, in some cases, there are alternative therapeutic options or equivalent medications where fewer side effects, such as drug-induced PLD, can be achieved.

Due to its adverse effects in vitro and presumably in vivo, PLD has drawn increasing amounts of attention, especially within the early stages of drug design[8a], [24] and drug approval and registration, even by the FDA.[25] Unfortunately, the available experimental data on PLD-inducing drugs is inadequate, unsatisfactory, and inhomogeneous. Several prominent studies have been published, including reports by Pelletier et al.[26] (who compiled experimental data for a set of 201 compounds), by Kruhlak et al.[25a] (who investigated 482 substances), and most recently, by van de Waters et al.[27] (who published data for a set of 56 compounds). In the past, various experimental methods have been proposed to measure PLD using cell-free,[28] in vitro,[29] and in vivo[30] approaches. Additionally, in silico prediction of PLD has been attempted in various studies using random forests,[31] support vector machines,[26], [31] nearest neighbor classifications,[32] decision trees,[32]–[33] logistic regressions,[26] Bayesian models,[26] and artificial neural networks,[32] based on the hitherto available data. Although some of these models are able to predict with reasonable accuracy, the low amount of available experimental data still limits the achievable performance. Accurate in silico prediction of drug-induced PLD could help to eliminate drug candidates showing PLD at an early stage of the drug design process. In the present study, we have significantly extended the amount of published experimental data on the PLD-inducing activity of previously untested compounds and constructed a prediction system based on molecular properties.

## Results

### Characterization of PLD by phospholipid content

**Figure 1:**
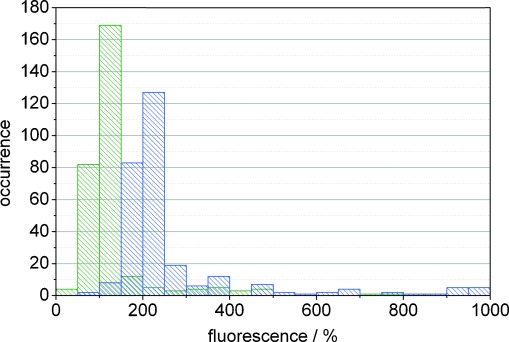
Histogram of measured cellular phospholipid fluorescence at both compound concentrations (green: 2.5 μm; blue: 5.0 μm) was used to determine the threshold for PLD-inducing agents. The results are given as a percentage (*x*-axis) of the corresponding control values. Assuming a normal distribution of the values resulting from inactive compounds with a mean of approximately 100 %, this plot suggests a limit of 200 % for active compounds, which is equal to a doubling of the LipidTox content.

**Figure 2:**
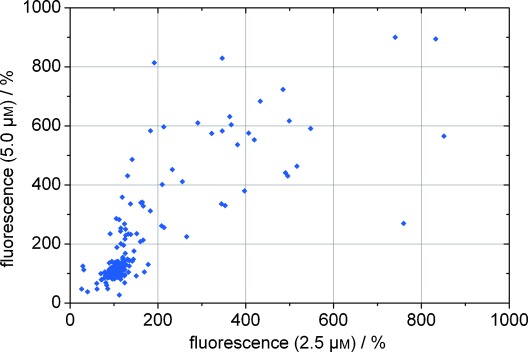
Scatterplot showing the experimentally determined cellular LipidTox fluorescence values given as a percentage of the respective control at 5.0 μm (*y*-axis) and 2.5 μm (*x*-axis). Most test compounds show an increase in phospholipid fluorescence with increasing concentration.

To determine the amount of cellular LipidTOX accumulation, which represents PLD, we displayed the results of our cell culture data for both tested concentrations in a histogram, shown in Figure 1. Most agents tested did not significantly influence the lysosomal metabolism of phospholipids compared with untreated control cells. Taking into account the mean standard deviation of the experimental values (11.1 % at 2.5 μm, 12.8 % at 5 μm) and the mean coefficient of variation (0.077 at 2.5 μm, 0.080 at 5 μm), a doubling of the cellular LipidTOX concentration is sufficient to identify PLD-inducing compounds. Therefore, we set 200 % as the threshold to classify a compound as a PLD-inducing agent. Applying this criterion, we identified 30 compounds as PLD-inducing agents at a concentration of 2.5 μm. At a concentration of 5.0 μm, 55 substances increased cellular LipidTOX content to values over 200 %. All compounds that induced PLD at 2.5 μm were also classified as PLD-inducing agents at the higher concentration of 5.0 μm (Figure 2). The maximal observed increase of lysosomal phospholipid content was induced by tamoxifen (**277**; 740.5 % at 2.5 μm, 900.2 % at 5.0 μm; Figure 2). Phospholipid fluorescence was elevated at the higher concentration for 46 of 55 PLD inducers. On average, the effect increased by 178.1±76.4 % when doubling the concentration from 2.5 μm to 5.0 μm, which suggests an overall dependence of PLD on drug concentration. Furthermore, the applied concentrations did not reduce the cell number significantly, as confirmed by 4′,6-diamidino-2-phenylindole (DAPI) staining.

### Identification of PLD-inducing agents

Altogether, 199 of the 297 compounds had not been previously tested for their effects on phospholipid metabolism. Of these 199 compounds, 20 induced PLD at 2.5 μm, and 28 induced PLD at 5.0 μm. These 28 compounds were not previously reported to induce PLD: amlodipine (**15**), astemizole (**21**), bromperidol (**30**), carvedilol (**37**), dilazep (**60**), fendiline (**69**), fluphenazine (**78**), loperamide (**94**), norfluoxetine (**112**), compazine (**134**), raloxifene (**146**), suloctidil (**154**), triflupromazine (**164**), camylofine (**180**), AY-9944 (**196**), pimozide **202**), clemastine (**208**), penfluridol (**213**), paroxetine (**218**), sertindole (**225**), mibefradil (**227**), tomatidine (**232**), desloratadine (**248**), cepharanthine (**256**), connesine (**258**), chlorprothixene (**269**), clomiphene (**276**), and flupenthixol (**284**). The newly identified PLD-inducing agents include various commonly used and approved drugs, such as bromperidol (**30**), carvedilol (**37**), and penfluridol (**213**). All experimental results are shown in Figure 2. A complete list of all experimental results can be obtained from the Supporting Information. In vivo, PLD is highly dependent on the concentration of the drug, so the specific therapeutic concentration must always be taken into consideration when assessing the risk of drug-induced PLD.[34]

### Prediction of PLD by a random forest model

We used our experimental data on PLD (*n*=297, *c*=5.0 μm) to develop an in silico model to predict whether or not a compound would induce PLD. Using the workflow explained below, we collected 167 models featuring a nonvalidated accuracy of 100 %, based on three and four descriptors. To distinguish between these equivalent models, we validated them with a bootstrap algorithm (*n*=100, sample ratio=1.0). Table 1 shows the best models for three and four descriptors. The best validated prediction system with four descriptors yielded a validated accuracy of 86.3 %. Remarkably, the best prediction system based on three descriptors yielded a validated accuracy of 84.6 %.

**Table 1:** The most predictive three- and four-descriptor models for PLD prediction

Atts[a]	Descriptor names	Accuracy[b] [%]	
		nonval.	val.
4	GCUT_SMR_0, si_vsa_acc, ACDlog *P*-NO, si_QMINN	100	86.3
3	GCUT_SMR_0, ACDlog *P*-logWeight, neutral_Slog *P*	100	84.7

[a] Number of attributes.

[b] Nonvalidated (nonval.) and validated (val.) accuracy. Validated by bootstrapping (sample ratio=1.0; number of validations=100).

### Interaction of PLD-inducing agents

**Figure 3:**
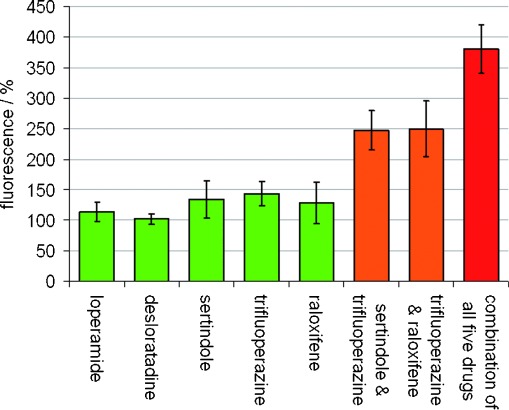
PLD is assumed to be caused by pharmacological interactions in an additive manner. Loperamide (**94**), desloratadine (**248**), sertindole (**255**), trifluoperazine (**163**), and raloxifene (**146**) at moderate concentrations (0.5 μm) only slightly affect cellular phospholipid levels, while combinations of these agents clearly induced PLD. Mean values are given ±SD.

To examine the effect of PLD-inducing agents administered in combination, we randomly selected PLD-inducing compounds from our test set and measured the effect of combinations of these compounds in low concentrations on cellular phospholipid levels. For this purpose, we selected five compounds that induced PLD at a final concentration of 5.0 μm (Figure 3): loperamide (**94**), desloraratadine (**248**), sertindole (**255**), trifluoperazine (**163**), and raloxifene (**146**). All five compounds induce PLD when applied at a concentration of 5.0 μm, but none of them duplicated these phospholipid levels at a concentration of 0.5 μm (loperamide: 114±16 %, desloraratadine: 102±9 %, sertindole: 134±30 %, trifluoperazine: 144±20 %, and raloxifene: 128±34 %). However, combinations of these agents each at 0.5 μm clearly increased cellular phospholipid levels. Even combinations of two agents at low concentration levels resulted in doubling of phospholipid levels (sertindole and trifluoperazine: 247±32 %, trifluoperazine and raloxifene: 250±45 %). The combination of all five compounds each at 0.5 μm increased the cellular phospholipid concentration to approximately 380±40 % compared with untreated control cells.

### Association between PLD and drug characteristics

Compounds must be able to pass through two biological membranes, namely the cellular membrane and the lysosomal membrane, to induce PLD. Thus, we compared agents that induce PLD to other biological targets in the context of membrane permeability and lysosomal metabolism.

**Figure 4:**
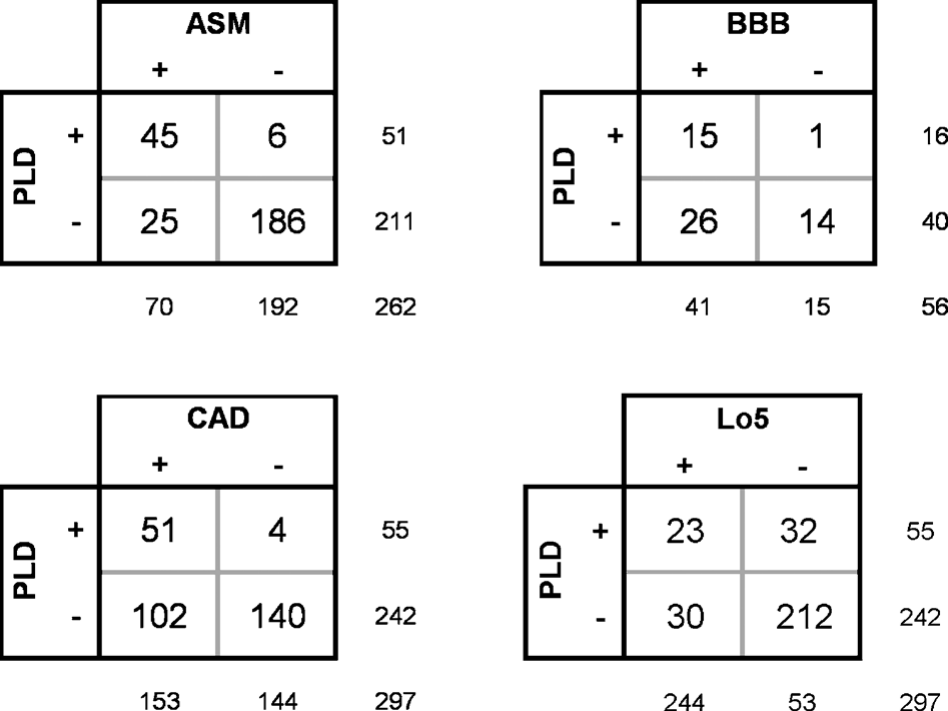
Associations between drug-induced PLD and properties related to membrane permeability (inhibition of ASM, BBB permeability, CAD characteristic, and Lo5 violations) were analyzed using contingency tables. The classification “+” represents PLD-inducing agents, ASM inhibitors, CAD (according to their molecular properties), and compounds that violate Lo5.

**Acid sphingomyelinase:** The acid sphingomyelinase (ASM) is an enzyme involved in the lipid metabolism of the lysosome and is relevant for lysosomal storage diseases in general. In a recent study,[35] 262 of the compounds tested here were also tested for their potential to inhibit ASM. Most of these compounds were either active or inactive for both targets, which is also supported by the χ^2^ likelihood ratio test (χ^2^=122.4, df=1, *p* <0.001). A contingency table is shown in Figure 4.

**Blood–brain barrier:** Another important property for drugs is blood–brain barrier (BBB) permeability. An association between BBB permeability and PLD can be assumed, as it also involves the penetration of at least two membrane systems, namely the luminal and abluminal plasma membrane of the endothelial cells in the brain. To examine the association between PLD and BBB permeability, we looked at BBB permeability measurements taken from a recent work.[36] Of all of the PLD-inducing agents with reported BBB permeability, only tacrine (**7**) was reported to have a slightly negative logBB value of −0.12.[37] However, other studies have concluded that **7** readily crosses the BBB,[38] which is in agreement with its use as an antidementia drug. As the contingency shows a low number of cases per class, Fisher’s exact test is recommended instead of the χ^2^ test. The Fisher’s exact test indicated statistical dependence of the two properties (*p*=0.043). A contingency table is shown in Figure 4.

**Cationic amphiphilic drugs:** Using our definition of CADs we classified all compounds as CAD or non-CAD based on their calculated log *P* and p*K*_a_ values given by ACD/Labs 10.0 (p*K*_a_>7.4, log *P*>3; see Experimental Section).[39] In total agreement with the requirements for cationic character, all PLD-inducing agents identified here feature a protonated basic center with an average highest basic p*K*_a_ of 9.04±1.38 and an average log *P* of 5.08±1.53. Only four of the 55 PLD-inducing agents were not classified as CADs, based on calculated p*K*_a_ and log *P* values and the cut-off values described in the Experimental Section (see Figure 4 for a contingency table). Thus, we took a closer look at those compounds. Perphenazine (**127**) and fluphenazine (**78**) have calculated p*K*_a_ values of 6.85 according to ACD/Labs, but an experimental p*K*_a_ of 7.9 has been reported for both of these two compounds.[40] The same situation occurs for flupenthixol (**284**) and amodiaquine (**16**), which both have an experimental p*K*_a_ value that is slightly above 7.4.[40], [41] The contingency shows a low number of cases for at least one class; Fisher’s exact test was applied instead of the χ^2^ test. The Fisher’s exact test indicates an association between the two properties (*p* <0.001). Thus, the combination of p*K*_a_ and log *P* (calculated or, preferably, experimental) can serve to predict PLD induction for a given compound.

**Lipinski’s rule of five:** A prominent artificial estimation of bioavailability is Lipinski’s rule of five (Lo5).[42] This rule was developed to distinguish between drug-like and non-drug-like compounds. Compounds that violate the Lo5 are considered to be less drug-like. We therefore analyzed the distribution of the tested compounds with respect to Lo5 violations. The χ^2^ likelihood ratio test indicates an association of the two properties (χ^2^=26.5, df=1, *p* <0.001). Thus, violation of Lo5 is also associated with PLD induction (contingency table shown in Figure 4).

### Enrichment of PLD-inducing drugs in ATC classes

One of the most commonly used classification systems for drugs with respect to their clinical applications is the anatomical therapeutic chemical (ATC) system,[43] which was developed by the World Health Organization (WHO). We used its second-level code to classify our compounds according to their respective fields of clinical application. We observed that certain ATC subgroups, such as N05, L02, P02, N06, and C08, show a high occurrence of PLD-inducing agents, whereas classes such as A03 barely contain any compounds that induce PLD. For a detailed overview, see Table 2. However, it should be noted that many PLD-inducing compounds share a similar scaffold, which might also contribute to their common classification by the ATC code.

**Table 2:** Distribution of PLD-active and PLD-inactive compounds with respect to their ATC classifications. Only classes that include at least one active and inactive compound are listed

ATC[a]	ATC group name	Active[b]	Inactive[b]	Ratio[c]
A03	Agents for functional gastrointestinal disorders	1	10	0.09
C01	Cardiac therapy	2	5	0.29
C04	Peripheral vasodilators	1	4	0.20
C07	Beta-blocking agents	1	3	0.25
C08	Calcium channel blockers	4	5	0.44
D04	Antipuritics including antihistamines, anesthetics, etc.	1	4	0.20
G03	Sex hormones and modulators of the genital system	2	7	0.22
J01	Antibacterials for systemic use	1	3	0.25
L02	Endocrine therapy	1	1	0.50
N05	Psycholeptics	13	10	0.57
N06	Psychoanaleptics	11	13	0.46
P01	Antiprotozoals	2	2	0.50
R06	Antihistamines for systemic use	3	6	0.33

[a] ATC short code.

[b] Compounds able to induce PLD at 5.0 μm were considered active.

[c] Ratio of active/all compounds in the corresponding ATC class.

## Discussion

We have studied the effects of a large number of small drug-like compounds on phospholipid metabolism. Many of these agents had not previously been tested for PLD-inducing effects; among them, we identified 28 novel PLD-inducers. Furthermore, we identified important characteristics of PLD-inducing drugs, namely their association with cationic amphiphilic properties, functional inhibition of ASM, BBB permeability, and with violations of Lo5. Finally, we developed a qualitative structure–property activity relationship model to predict PLD induction with reasonable validated accuracy, which we will discuss critically with respect to several topics.

### LipiTox as a versatile method to measure PLD

PLD causes extensive accumulation of phospholipids within the lysosome, leading to changes in the lysosomal morphology, such as the formation of multilamellar bodies.[21] The presence of multilamellar bodies was formerly used to identify PLD-inducing agents in vitro. This visualization requires electron microscopy.[19b] Because electron microscopy is time-consuming and expensive, this technique is not useful for fast and broad screening.[3a], [44] The synthesis of phospholipids that are stably labelled with a fluorescent tag and the development of automated fluorescence readouts enabled the design of a reliable high-throughput assay (HTA) to identify PLD-inducing compounds.[29b] This HTA is suitable for determining the lysosomal aggregation of phospholipids upon application of chemical compounds in a fast, reproducible, and quantitative manner. In contrast to previously described HTAs for determining drug-induced PLD,[23], [29b] we employed the adherent, rapidly proliferating, human neuroglioma cell line H4, as this neuroglioma cell line is perfectly compatible with the LipidTOX assay. The H4 neuroglioma cell line that was used here exhibits a high proliferation rate (24 h per cell cycle).[45] Additionally, H4 cells feature remarkable robustness, even when incubated with high drug concentrations. We want to emphasize that LeCureux and colleagues were able to show drug-induced PLD for several cell lines and even primary macrophages.[46] This suggests that similar results can be obtained independently from the setup of this assay.

### Therapeutic concentrations of the tested drugs

Typical therapeutic concentrations for common drugs range from below 1.0 μm up to approximately 5.0 μm,[34b],c, [47] but their tissue concentrations can be much higher due to phenomena such as lysosomal trapping.[41], [48] To mimic the in vivo situation, we tried to apply the test compounds within the range of their therapeutic plasma concentrations. With respect to the homogeneity of our results, we tested all compounds at concentrations of 2.5 μm and 5.0 μm.[49]

### PLD has no acute effect on the cell count

**Figure 5:**
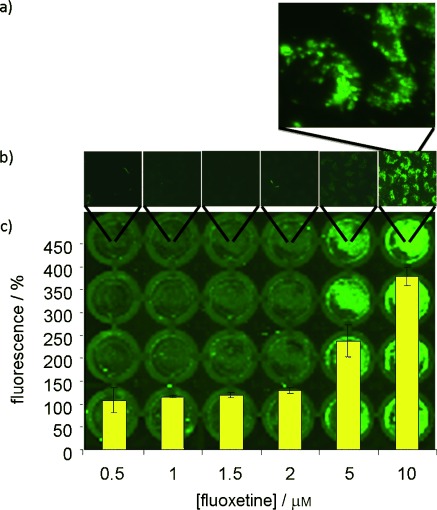
Cellular LipidTox content increases upon incubation of cells with fluoxetine (**79**). Human neuroglioma cells (H4) were treated with the well-known PLD inducer fluoxetine in the presence of LipidTox. Each concentration was applied in quadruplicate, and bars represent the mean values of LipidTox fluorescence ±SD. To further validate our results (c), representative pictures obtained with a fluorescence microscope are shown (b). The enlarged fluorescence microscope images highlight the punctual localization of the fluorescent phospholipid mixture (a).

Independently of PLD, toxic drug effects can cause a reduction in the number of cells, and subsequently in the fluorescence signal, so a method for monitoring the number of cells in addition to the cellular LipidTOX content is required. As dead cells do not take up LipidTOX, induction of PLD by the cytotoxic effects of compounds could not be measured by the cell-culture-based assay described here. To exclude this possibility, we employed the well-established fluorescent cell stain DAPI,[50] which has previously been used in other automated cell counting applications.[51] Our results demonstrate that staining with the fluorescent nuclear dye DAPI is appropriate for quantifying the number of cells (Figure 5). As described above, DAPI fluorescence of the cells showing PLD is comparable to that of the controls.

### Selection of descriptors to predict PLD

The random forest prediction system for three and four descriptors consists mainly of descriptors already known to be relevant to membrane permeability and bioavailability. The four-descriptor model includes the surface area of hydrogen bond acceptors (si_vsa_acc), a descriptor derived from log *P* (ACDlog *P*-NO), accounting for the minimal charge at the most basic nitrogen atom (QMINN) and the atomic contributions to molar refractivity (GCUT_SMR_0). Although the contribution of this last descriptor cannot be easily explained by any physicochemical relationships, it appears in the majority of highly predictive models. The three-descriptor model also contains GCUT_SMR_0, along with two descriptors derived from log *P* (ACDlog *P*-logWeight, neutral_Slog *P*). Together, the descriptors selected for the best models here suggest that physicochemical properties related to membrane permeability and charge seem to be most useful for the prediction of PLD.

### Comparison with previous experimental results

We compared our experimental results with data from previously published studies by Lowe et al.,[31] Kruhlak et al.,[25a] Hanumegowda et al.,[52] Pelletier et al.,[26] and van de Water et al.[44b] Experimental values for 98 of the compounds tested in this study were found in these references. We excluded 7 compounds that had conflicting results from the different references, which left us with published data for 91 compounds. Our results for 60 of these compounds were in agreement with previously reported findings in the literature. Thus, compounds such as amitriptyline (**14**) and amiodarone (**12**) were consistently found to induce PLD across different experimental settings and conditions. However, our experimental results for 31 compounds were not in agreement with previously published results. Due to the relatively low concentrations of the compounds used in our experiments, we may have mainly identified agents that strongly induce PLD. Only perphenazine (**127**) had a previously reported negative result but was found to induce PLD in this study.

Altogether, the number of compounds with different results between this and other studies highlights the importance of the experimental setting, including factors such as drug concentration. For example, van de Water et al.[44b] performed experiments at various concentrations ranging from 0.3 μm to 31.6 μm. Other studies have compiled datasets from literature observations. In contrast, the dataset presented here stands out due to its homogenous and uniform experimental setting, which should produce directly comparable results.

### Comparison with previous binary classification systems

We also compared our in silico model to other previously published prediction systems to benchmark and rate the performance of the current system. All published models classified the drugs in a binary manner with respect to PLD induction and used the accuracy to evaluate the performance of the model, which enables comparison of the models. Although the published models differed in terms of the method, number of descriptors, and number of compounds (in the training set), the accuracy can be calculated to compare the performance of all models. Table 3 summarizes the performance of these previously published models.

**Table 3:** Overview of previously published in silico models for predicting PLD induction. Taking into account the number of tested compounds and the number of descriptors used, our results outperform the previously published models and also suggest that random forest models are well-suited for predicting PLD activity

Descriptors[a]	Compds	Accuracy[b] [%]		Method	Ref.
		nonval.	val.		
Clog *P*, p*K*_a_, atom and ring counts	450	76.4	74.6	DT	[33a]
SMARTS patterns	450	87.6	88.1	RB	[33a]
Clog *P*, 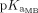 , *V*_d_	101	88.1	–	LDA	[52]
ΔΔ*G*_AM,_ p*K*_a_, Clog *P*	32	–	90.6	RB	[53]
log *P*, p*K*_a_, amphipilic momentum	201	83.1	–	BM	[26]
log *P*, SMR, vsa_acc, QMINN	297	100	86.3	RF	*this work*

[a] Abbreviations: Bayesian model (BM), decision tree (DT), linear discriminant analysis (LDA), random forest (RF), rule-based decision (RB).

[b] Nonvalidated (nonval.) and validated (val.) accuracy.

Our work outperformed all other models in terms of nonvalidated accuracy. In terms of validated accuracy, Fischer et al.[53] achieved comparable accuracy with a slightly smaller validation set. We want to stress that the validated accuracy is also dependent on the validation method.

### Mechanisms responsible for the induction of PLD

Five different mechanisms for the induction of PLD by chemical compounds have been proposed thus far:[12] 1) CADs bind to phospholipids and prevent their degradation by lysosomal phospholipases;[54] 2) CADs stimulate phospholipid synthesis in the cell;[55] 3) CADs bind to lysosomal phospholipases and inhibit enzymatic activity by allosteric or competitive mechanisms;[56] 4) CADs reduce lysosomal homing of lysosomal phospholipases by inhibiting mannose-6-phosphate receptor-mediated lysosomal sorting;[57] 5) CADs displace lysosomal phospholipases from the lysosomal membrane,[18] and subsequent degradation by lysosomal proteases causes a reduction in cellular phospholipase activity.[58]

The final mechanism listed above is similar to the mechanism for functional inhibition of lysosomal ASM by CADs. In its active state, ASM is attached to the inner lysosomal membrane by an electrostatic interaction.[18], [59] The inner lysosomal membrane is negatively charged due to its most abundant phospholipid, bis-(monoacylglycero)-phosphate (BMP).[60] Positively charged proteins such as ASM are attached by electrostatic interactions to the lysosomal membrane[18] and are subsequently protected from lysosomal degradation.[58] The incorporation of positively charged compounds, such as CADs, into the negatively charged lysosomal membranes could lower the effective charge and weaken the electrostatic membrane–protein interactions. Therefore, active enzymes might detach from the membrane and be degraded by proteolysis within the lysosome.[58] According to this mechanism, inhibition of lysosomal enzymes may be seen not as a direct effect of CADs but rather a functional one[61] that needs to be analyzed and proven by membrane–protein interaction studies in vitro.[2], [18], [62] Due to the high overlap between ASM inhibitors and PLD inducers, we suspect that ASM and lysosomal phospholipases are inhibited by CADs in a similar fashion. This hypothesis is further supported by a recent publication confirming the ionic attachment of lysosomal phospholipase A_2_ to negatively charged membranes, which was weakened by amiodarone (**12**).[63] Also in agreement with our hypothesis, the in vitro activity of lysosomal phospholipase A_1_ increases when higher amounts of negatively charged lipids are added to the reaction.[64]

A functional mechanism would also be in agreement with the results from experiments on combinations of PLD inducing agents, as PLD appears to be triggered by molecular properties rather than by specific protein–drug interactions.

### Strengths and limitations

The present study used a consistent and reproducible method to measure drug-induced PLD for an immortalized, quickly proliferating cell line. This fluorescence-based assay would facilitate the high throughput screening of compounds. However, the results of this assay cannot be compared with a tissue-specific situation in vivo without careful consideration, although several studies show comparable results.[46], [65]

The in silico model presented here is appropriate for analyzing only compounds that are similar to the tested compounds. This model should therefore be used for drug-like compounds. Anionic compounds, for example, are largely underrepresented in the tested set and might lead to incorrect predictions.

## Conclusions

In this study, we present experimental in vitro data of drug-induced PLD for 297 drug-like compounds at two different concentration levels (2.5 μm and 5.0 μm). Of these compounds, 206 had not been previously tested for their effects on phospholipid metabolism. We identified 20 novel PLD-inducing agents at 2.5 μm and 28 novel PLD-inducing agents at 5.0 μm. PLD-inducing compounds seem to share specific molecular requirements, as they show a high concordance with agents that have similar biological targets, such as ASM inhibitors and BBB-permeable compounds. We also found an association between numerical estimations for bioavailability, such as classification as CADs or violation of Lo5, and PLD-inducing compounds. There is an additive effect for PLD-inducing compounds when applied in combination. Therefore, combinations of PLD-inducing drugs should be used for clinical applications with caution.

## Experimental Section

**Cell culture**: Human brain neuroglioma H4 cells were purchased from Promochem (Wesel, Germany). The cells were cultivated in Dulbecco’s modified Eagle’s medium (Biochrom, Berlin, Germany) supplemented with 10 % (*v*/*v*) fetal bovine serum (FBS) and 4 mm glutamine (all from Biochrom, Berlin, Germany). The cell line was maintained at 37 °C in a humidified atmosphere containing 8.5 % CO_2_ and was routinely split at a ratio of 1:6. The cells were regularly tested for mycoplasma contamination by an ELISA-based assay (Lonza, Basel, Switzerland), and the tests were always negative.

**Compound library**: All substances were purchased from Sigma–Aldrich (Hamburg, Germany), Biotrend (Cologne, Germany), Chemos (Regenstauf, Germany), Tocris (Bristol, UK), Aurora Fine Chemicals (Graz, Austria), or AstraZeneca (London, UK) at the highest purity available. All chemicals were dissolved in water, DMSO, EtOH, or MeOH at 10 mm each and were stored at −20 °C. To sterilize the dissolved compounds, each solution was filtered (0.45 μm pore size). Each substance received a number, and all of the experiments were conducted in a blind manner to avoid any form of bias. In total, we selected 297 small drug-like compounds for testing. Basic lipophilic compounds were overrepresented to further investigate the relevance of CADs to PLD. A complete list of all of the compounds can be found in the Supporting Information.

**Quantification of drug-induced PLD**: H4 cells were seeded in 96-well white-well dishes (Nunc, Langenselbold, Germany) at a density of 4×10^3^ cells per well. After 48 h, the medium was replaced with fresh medium that included the test substances and HCS LipidTOX Green phospholipidosis detection reagent (Invitrogen, San Diego, US) at their respective final concentrations. Each test substance was diluted from the stock solution with medium and was applied at 2.5 μm and 5.0 μm for an additional 24 h. All tests were performed in quadruplicate. During the test period, the cells were kept at 37 °C in a humidified atmosphere containing 8.5 % CO_2_. To quantify the lysosomal phospholipid content, the cells were washed with phosphate-buffered saline (PBS), counterstained with DAPI (Carl Roth GmbH, Karlsruhe, Germany) and fixed with 10 % neutral buffered formalin (AppliChem GmbH, Darmstadt, Germany). Fluorescent signals were counted with a fluorescence reader (Perkin Elmer, Waltham, US) at an excitation wavelength of 485 nm and an emission wavelength of 535 nm for the lysosomal dye (LipidTOX Green) and at an excitation wavelength of 355 nm and an emission wavelength of 470 nm for the nuclear dye (DAPI). Results were corrected by subtraction of the background and are given in percent fluorescence of the corresponding control averaged over four experiments.

The PLD LipidTox assay was tested using the well-known PLD-inducing drug fluoxetine (**79**)[20c] at various concentrations to optimize the experimental settings. A nearly linear relationship between lysosomal LipidTOX fluorescence and **79** concentration was obtained from 1 μm to 10 μm (Figure 5 a). This correlation is highlighted by fluorescence microscope images of cells treated with **79** and the corresponding quantification of this fluorescence (Figure 5 a). Together, this confirms that the fluorescent phospholipid mixture that we used enabled quantitative evaluation of drug-induced PLD. The fluorescence microscope images shown in Figure 5 b demonstrate that the fluorescence values given in Figure 5 c are due to fluorescent cells which absorbed the fluorescent phospholipid mix LipidTOX upon treatment with **79**. Furthermore, the punctuated cellular LipidTOX localization in Figure 5 a confirms the accumulation of LipidTOX in cellular lysosomes upon induction of PLD by **79**.

**Figure 6:**
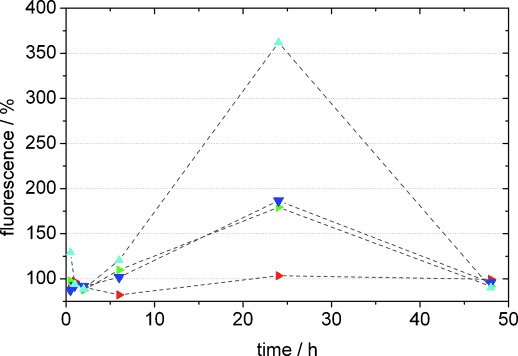
The optimal time to examine drug-induced PLD in our cell-culture-based assay was 24 h after addition of LipidTox. Experiments performed at different incubation times for four compounds with slow lysosomal accumulation (dicyclomine (**59**): 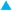; bepridil (**25**): 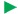; lofepramine (**93**): 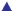; solasodine (**259**): 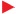) suggest that this is the optimal incubation time. LipidTox was added 24 h before the indicated time points. Results are given as mean values of quadruplicate measurements compared with the respective control.

**Lysosomal accumulation kinetics**: It is evident that drugs must enter both the cell and the lysosome to facilitate PLD.[12] It has already been shown that lysosomotropic drugs with high log *P* and p*K*_a_ values exhibit relatively slow lysosomal accumulation kinetics.[66] To ensure that the incubation time was sufficient for all compounds to achieve equilibrated lysosomal concentrations, including those with a slow rate of lysosomal accumulation, we conducted experiments with various compound incubation times ranging from 30 min to 48 h at a constant concentration of 5 μm. For this experiment, we selected three known PLD-inducing compounds (bepridil **25**, lofepramine **93**, and solasodine **259**) with slow lysosomal accumulation kinetics as suggested by a prediction model.[67] As Figure 6 shows, these three compounds exhibit maximal lysosomal phospholipid concentrations after an incubation time of 24 h. Cells treated for 6 h and 48 h did not show significant differences in their phospholipid load compared with untreated control cells. Dicyclomine (**59**) did not increase the lysosomal phospholipid content to values greater than 135.7±10.2 % of the untreated control cells at any tested incubation time and was therefore used as a negative control. Due to the rapid proliferation rate of the H4 cell line, the phospholipid and drug content is split between the daughter cells at every cell division (24 h). Reduction of phospholipid and drug content caused by cell division would therefore counteract PLD-inducing drug effects. Experimental data confirm this effect, as the extent of cellular LipidTox fluorescence decreased at longer incubation times for all compounds tested (see Figure 6). Hence, we reasoned that a compound incubation time of 24 h is sufficient to guarantee equilibrated lysosomal concentrations within the applied cell culture approach.

**Figure 7:**
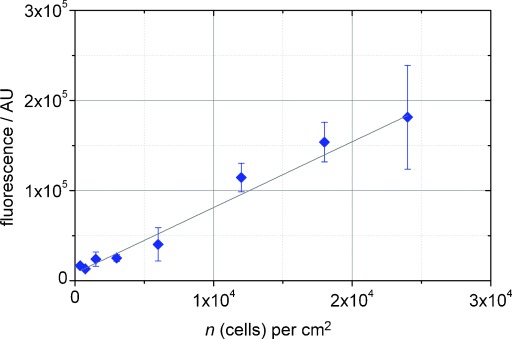
DAPI staining is a valid method for demonstrating the presence of cells in a high-throughput manner. H4 cells were seeded at different cell densities (*n*) as indicated. Quantified DAPI fluorescence clearly shows a correlation between measured fluorescence and the number of cells seeded up to 20 000 cells per cm^2^. This linear relationship enables confirmation of the presence of cells in high-throughput PLD assays. Mean values are given ±SD.

**Monitoring of cell numbers by nuclear staining**: When observing cellular responses to chemical compounds, it is important to also ensure the survival of cells upon treatment with the test agent. Because we are assuming comparable cell numbers for each experiment (including the untreated controls), toxic drug effects or reduced cellular adhesion would distort the results and render them incomparable. Thus, the nuclei of the cells were stained with the fluorescent nuclear dye DAPI. To confirm the correlation between DAPI fluorescence and cell number, we seeded increasing numbers of cells and stained the cells with DAPI 48 h later. The applied DAPI stain appears to be a suitable method for quantifying the number of adherent cells within cell culture jars (Figure 7). Our results demonstrate that DAPI fluorescence increased linearly with cell number until 2.4×10^4^ cm^2^ (see Figure 7). On average, none of the tested compounds reduced the cell number to a mean value below 94.0±34.5 % of the untreated controls at any concentration tested. Additionally, agents that induced PLD did not significantly reduce DAPI fluorescence as compared with compounds that did not increase the cellular LipidTOX content (see Table 4). Therefore, we conclude that the conditions used in the assays do not cause an acute loss of cells.

**Table 4:** Average DAPI fluorescence relative to the respective controls. Data show that systematic reduction in cell numbers is not associated with PLD induction

Set	*n*	Fluorescence[a] (2.5 μm) [%]	*n*	Fluorescence[a] (5.0 μm) [%]
PLD-inducing agents	55	93.5±23.8	30	86.9±39.8
Inactive agents	242	99.1±43.2	267	95.6±33.1
Total	297	98.1±40.4	297	94.0±34.5

[a] Average DAPI fluorescence at 2.5 μm or 5.0 μm as a percent of untreated controls.

**Calculation of descriptors**: Structures for all compounds were obtained from the PubChem Database as SDF files. We calculated molecular descriptors provided by MOE2010.10[68] and ACD/Labs 10.0,[39] two widely used software packages that cover a broad range of molecular, topological, and physicochemical properties. A relationship between CADs and PLD has been proposed in many published reports.[10], [12] As there is still no clear definition of CADs based on molecular properties, we suggest that the cationic character of a molecule be characterized by its highest basic p*K*_a_ value and the amphiphilicity of a charged molecule be characterized by its log *P* value. Our definition is in accordance with previous studies showing that the most acidic p*K*_a_ value,[69] the highest basic p*K*_a_ value, and the log *P* value highly influence the chance that a molecule will induce PLD.[33b]

* Cationic is defined as a positively charged compound. CADs must have at least one positively charged atom and a positive overall charge. Typically, charged compounds at physiological pH have a highest basic p*K*_a_>7.4.
* Amphiphilic compounds require both hydrophilic and hydrophobic portions in their structures. A charged species is inherently hydrophilic, which leads to a lower log *P* value, so a charged molecule that still has a high log *P* value would fulfill the criteria for being amphiphilic. We suggest that a log *P*>3.0 should be sufficient to guarantee amphiphilic character for a cationic compound.

**Calculation of statistic measures**: The statistic measures used in this work were calculated using the R statistics software (version 2.15.0). For the χ^2^ statistic, which was calculated without Yates correction, *p* values below 0.05 were considered significant. For small or unbalanced data with at least one classification occurring <5, we used Fisher’s exact test instead of the χ^2^ test, as recommended in the literature.[70] Mean values are given with their corresponding standard deviations (SD).

**Development of the prediction model**: We selected predictive descriptors using feature selection algorithms (provided by RapidMiner 5.1.13), which returned 113 descriptors as presumably predictive. A beam search was used with this filtered dataset (width=number of descriptors=113) to select the most predictive combination of descriptors.[71] For every combination, a random forest (ntrees=5, treedepth=8) was applied, and the corresponding accuracy was calculated as a fitness criterion. As a random forest depends on the random seed used, we performed the workflow multiple times with different random seeds. The beam search stopped whenever an accuracy of 1.00 was returned, as this is the maximal accuracy. These models were then validated by bootstrapping (*n*=100, sample ratio=1.0) to reveal the optimal validated model for PLD prediction.

## Supplementary material

Detailed facts of importance to specialist readers are published as ”Supporting Information”. Such documents are peer-reviewed, but not copy-edited or typeset. They are made available as submitted by the authors.
