# Supplementary material for: Identification of Drugs Inducing Phospholipidosis by Novel in vitro Data
Source: ChemMedChem. 2012 Sep 3;7(11):1925–34. doi: 10.1002/cmdc.201200306 (PMC3533795; doi:10.1002/cmdc.201200306)
Supplement: Supplementary file 1 [file cmdc0007-1925-SD1.pdf]

CHEMMEDCHEM
Chemistry & Drug Discovery

Supporting Information

© Copyright Wiley-VCH Verlag GmbH & Co. KGaA, 69451 Weinheim, 2012

Identification of Drugs Inducing Phospholipidosis by Novel in vitro Data

Markus Muehlbacher,[a, b] Philipp Tripal,[a] Florian Roas,[a] and Johannes Kornhuber*[a]

cmdc_201200306_sm_miscellaneous_information.pdf

#	CID	Name	Supplier	SMILES	PLD (2.5µM)	PLD (5.0µM)
1	237	quinacrine	Sigma	CCN(CC)CCCC(C)NC1=C2C=C(C=CC2=NC3=C1C=CC(=C3)Cl)OC	516.4	463.6
2	444	bupropione	Sigma	CC(C(=O)C1=CC(=CC=C1)Cl)NC(C)(C)C	108.9	99.6
3	681	3-hydroxytyramine	Sigma	C1=CC(=C(C=C1CCN)O)O	125.0	96.1
4	896	melatonin	Sigma	CC(=O)NCCC1=CNC2=C1C=C(C=C2)OC	88.8	102.8
5	1691	doxorubicin	Tocris	CC1C(C(CC(O1)OC2CC(CC3=C(C4=C(C(=C23)O)C(=O)C5=C(C4=O)C=CC=C5OC)O)(C(=O)CO)O)N)O	26.3	47.4
6	1775	phenytoin	Sigma	C1=CC=C(C=C1)C2(C(=O)NC(=O)N2)C3=CC=CC=C3	109.7	97.9
7	1935	tacrine	Sigma	C1CCC2=NC3=CC=CC=C3C(=C2C1)N	366.8	604.1
8	2094	allopurinol	Sigma	C1=C2C(=NC=NC2=O)NN1	72.3	79.5
9	2119	alprenolol	Sigma	CC(C)NCC(COC1=CC=CC=C1CC=C)O	101.7	98.5
10	2130	amantadine	Sigma	C1C2CC3CC1CC(C2)(C3)N	96.6	94.7
11	2132	ambroxol	Sigma	C1CC(CCC1NCC2=CC(=CC(=C2N)Br)Br)O	112.2	125.7
12	2157	amiodarone	Sigma	CCCCC1=C(C2=CC=CC=C2O1)C(=O)C3=CC(=C(C(=C3)I)OCCN(CC)CC)I	346.2	583.0
13	2159	amisulpride	Sigma	CCN1CCCC1CNC(=O)C2=CC(=C(C=C2OC)N)S(=O)(=O)CC	90.5	90.8
14	2160	amitriptyline	Sigma	CN(C)CCC=C1C2=CC=CC=C2CCC3=CC=CC=C31	114.9	243.5
15	2162	amlodipine	Sigma	CCOC(=O)C1=C(NC(=C(C1C2=CC=CC=C2Cl)C(=O)OC)C)COCCN	213.1	596.7
16	2165	amodiaquine	Sigma	CCN(CC)CC1=C(C=CC(=C1)NC2=C3C=CC(=CC3=NC=C2)Cl)O	353.0	330.0
17	2168	amorolfine	Biotrend	CCC(C)(C)C1=CC=C(C=C1)CC(C)CN2CC(OC(C2)C)C	96.7	106.8
18	2200	antazoline	Sigma	C1CN=C(N1)CN(CC2=CC=CC=C2)C3=CC=CC=C3	108.7	89.2
19	2215	apomorphine	Tocris	CN1CCC2=CC=CC3=C2C1CC4=C3C(=C(C=C4)O)O	105.1	139.9
20	2218	aprindine	VWR	CCN(CC)CCCN(C1CC2=CC=CC=C2C1)C3=CC=CC=C3	89.5	109.0
21	2247	astemizole	Sigma	COC1=CC=C(C=C1)CCN2CCC(CC2)NC3=NC4=CC=CC=C4N3CC5=CC=C(C=C5)F	759.6	269.5
22	2318	benofluorex	Sigma	CC(CC1=CC(=CC=C1)C(F)(F)F)NCCOC(=O)C2=CC=CC=C2	103.3	111.2
23	2331	benzamide	Sigma	C1=CC=C(C=C1)C(=O)N	98.2	94.0
24	2333	benzbromarone	Sigma	CCC1=C(C2=CC=CC=C2O1)C(=O)C3=CC(=C(C(=C3)Br)O)Br	88.3	99.7
25	2351	bepridil	Sigma	CC(C)COCC(CN(CC1=CC=CC=C1)C2=CC=CC=C2)N3CCCC3	125.4	168.2
26	2369	betaxolol	Sigma	CC(C)NCC(COC1=CC=C(C=C1)CCOCC2CC2)O	112.8	105.9
27	2381	biperidene	VWR	C1CCN(CC1)CCC(C2CC3CC2C=C3)(C4=CC=CC=C4)O	119.6	110.4
28	2442	bromhexine	Sigma	CN(CC1=CC(=CC(=C1N)Br)Br)C2CCCCC2	108.3	111.7
29	2446	bromopride	Sigma	CCN(CC)CCNC(=O)C1=CC(=C(C=C1OC)N)Br	105.1	95.2
30	2448	bromperidol	Sigma	C1CN(CCC1(C2=CC=C(C=C2)Br)O)CCCC(=O)C3=CC=C(C=C3)F	232.8	452.1
31	2474	bupivacaine	Sigma	CCCCN1CCCCC1C(=O)NC2=C(C=CC=C2C)C	100.7	100.9
32	2477	buspirone	VWR	C1CCC2(C1)CC(=O)N(C(=O)C2)CCCCN3CCN(CC3)C4=NC=CC=N4	102.2	108.4
33	2484	butenafine	Chemos	CC(C)(C)C1=CC=C(C=C1)CN(C)CC2=CC=CC3=CC=CC=C32	94.0	106.9
34	2520	verapamil	Sigma	CC(C)C(CCCN(C)CCC1=CC(=C(C=C1)OC)OC)(C#N)C2=CC(=C(C=C2)OC)OC	120.2	149.9
35	2554	carbamazepine	Sigma	C1=CC=C2C(=C1)C=CC3=CC=CC=C3N2C(=O)N	102.7	102.8
36	2562	carbetapentane	Sigma	CCN(CC)CCOCCOC(=O)C1(CCCC1)C2=CC=CC=C2	105.1	117.9
37	2585	carvedilol	Sigma	COC1=CC=CC=C1OCCNCC(COC2=CC=CC3=C2C4=CC=CC=C4N3)O	209.5	401.4
38	2719	chloroquine	Sigma	CCN(CC)CCCC(C)NC1=C2C=CC(=CC2=NC=C1)Cl	182.5	311.9
39	2725	chlorpheniramine	Sigma	CN(C)CCC(C1=CC=C(C=C1)Cl)C2=CC=CC=N2	90.5	102.2
40	2726	chlorpromazine	Sigma	CN(C)CCCN1C2=CC=CC=C2SC3=C1C=C(C=C3)Cl	125.0	217.1
41	2747	cibenzoline	Sigma	C1CN=C(N1)C2CC2(C3=CC=CC=C3)C4=CC=CC=C4	101.2	106.2
42	2769	cisapride	Sigma	COC1CN(CCC1NC(=O)C2=CC(=C(C=C2OC)N)Cl)CCCOC3=CC=C(C=C3)F	121.0	124.8
43	2771	citalopram	Sigma	CN(C)CCCC1(C2=C(CO1)C=C(C=C2)C#N)C3=CC=C(C=C3)F	88.6	96.9
44	2780	clebopride	Sigma	COC1=CC(=C(C=C1C(=O)NC2CCN(CC2)CC3=CC=CC=C3)Cl)N	104.0	94.0
45	2783	clenbuterole	Sigma	CC(C)(C)NCC(C1=CC(=C(C(=C1)Cl)N)Cl)O	91.9	97.9
46	2786	clindamycine	Sigma	CCCC1CC(N(C1)C)C(=O)NC(C2C(C(C(C(O2)SC)O)O)O)C(C)Cl	115.6	103.3
47	2794	clofazimine	Sigma	CC(C)N=C1C=C2C(=NC3=CC=CC=C3N2C4=CC=C(C=C4)Cl)C=C1NC5=CC=C(C=C5)Cl	177.7	130.7
48	2801	clomipramine	Sigma	CN(C)CCCN1C2=CC=CC=C2CCC3=C1C=C(C=C3)Cl	132.5	233.8
49	2803	clonidine	Biotrend	C1CN=C(N1)NC2=C(C=CC=C2Cl)Cl	93.0	101.3
50	2805	cloperastine	Sigma	C1CCN(CC1)CCOC(C2=CC=CC=C2)C3=CC=C(C=C3)Cl	120.4	116.3

51	2818	clozapine	Sigma	CN1CCN(CC1)C2=C3C=CC=CC3=NC4=C(N2)C=C(C=C4)Cl	105.4	107.6
52	2895	cyclobenzaprine	Sigma	CN(C)CCC=C1C2=CC=CC=C2C=CC3=CC=CC=C31	111.7	282.8
53	2898	cyclofenile	Sigma	CC(=O)OC1=CC=C(C=C1)C(=C2CCCCC2)C3=CC=C(C=C3)OC(=O)C	85.6	85.9
54	2905	cyclopentolate	Sigma	CN(C)CCOC(=O)C(C1=CC=CC=C1)C2(CCCC2)O	100.9	98.7
55	2913	cyproheptadine	Sigma	CN1CCC(=C2C3=CC=CC=C3C=CC4=CC=CC=C42)CC1	98.7	124.7
56	2995	desipramine	Sigma	CNCCCN1C2=CC=CC=C2CCC3=CC=CC=C31	118.7	358.3
57	3016	diazepam	Sigma	CN1C(=O)CN=C(C2=C1C=CC(=C2)Cl)C3=CC=CC=C3	103.0	111.0
58	3033	diclofenac	Sigma	C1=CC=C(C(=C1)CC(=O)O)NC2=C(C=CC=C2Cl)Cl	97.0	98.3
59	3042	dicyclomine	Sigma	CCN(CC)CCOC(=O)C1(CCCCC1)C2CCCCC2	89.1	135.7
60	3074	dilazep	Sigma	COC1=CC(=CC(=C1OC)OC)C(=O)OCCCN2CCCN(CC2)CCCOC(=O)C3=CC(=C(C(=C3)OC)OC)OC	137.3	335.8
61	3100	diphenhydramine	Sigma	CN(C)CCOC(C1=CC=CC=C1)C2=CC=CC=C2	78.3	85.4
62	3103	diphenylpyraldine	Sigma	CN1CCC(CC1)OC(C2=CC=CC=C2)C3=CC=CC=C3	111.4	112.2
63	3114	disopyramide	Sigma	CC(C)N(CCC(C1=CC=CC=C1)(C2=CC=CC=N2)C(=O)N)C(C)C	102.8	99.6
64	3152	donepezil	VWR	COC1=C(C=C2C(=C1)CC(C2=O)CC3CCN(CC3)CC4=CC=CC=C4)OC	103.5	116.3
65	3166	drofenine	Sigma	CCN(CC)CCOC(=O)C(C1CCCCC1)C2=CC=CC=C2	95.1	140.8
66	3168	droperidol	Sigma	C1CN(CC=C1N2C3=CC=CC=C3NC2=O)CCCC(=O)C4=CC=C(C=C4)F	100.6	94.4
67	3241	epinastine	Sigma	C1C2C3=CC=CC=C3CC4=CC=CC=C4N2C(=N1)N	101.3	93.1
68	3290	ethopropazine	Sigma	CCN(CC)C(C)CN1C2=CC=CC=C2SC3=CC=CC=C31	123.4	125.2
69	3336	fendiline	Sigma	CC(C1=CC=CC=C1)NCCC(C2=CC=CC=C2)C3=CC=CC=C3	105.1	286.3
70	3337	fenfluramine	Sigma	CCNC(C)CC1=CC(=CC=C1)C(F)(F)F	103.9	98.6
71	3339	fenofibrate	Sigma	CC(C)OC(=O)C(C)(C)OC1=CC=C(C=C1)C(=O)C2=CC=C(C=C2)Cl	86.2	96.7
72	3344	fenspiride	Sigma	C1CN(CCC12CNC(=O)O2)CCC3=CC=CC=C3	98.0	87.5
73	3348	fexofenadine	Sigma	CC(C)(C1=CC=C(C=C1)C(CCCN2CCC(CC2)C(C3=CC=CC=C3)(C4=CC=CC=C4)O)O)C(=O)O	80.7	92.8
74	3351	fipexide	Sigma	C1CN(CCN1CC2=CC3=C(C=C2)OCO3)C(=O)COC4=CC=C(C=C4)Cl	97.6	100.1
75	3354	flavoxate	Sigma	CC1=C(OC2=C(C1=O)C=CC=C2C(=O)OCCN3CCCCC3)C4=CC=CC=C4	93.9	99.7
76	3356	flecainide	Sigma	C1CCNC(C1)CNC(=O)C2=C(C=CC(=C2)OCC(F)(F)F)OCC(F)(F)F	96.0	81.7
77	3371	flufenamic	Sigma	C1=CC=C(C(=C1)C(=O)O)NC2=CC=CC(=C2)C(F)(F)F	81.3	97.3
78	3372	fluphenazine	Sigma	C1CN(CCN1CCCN2C3=CC=CC=C3SC4=C2C=C(C=C4)C(F)(F)F)CCO	363.2	631.2
79	3386	fluoxetine	Sigma	CNCCC(C1=CC=CC=C1)OC2=CC=C(C=C2)C(F)(F)F	141.3	486.4
80	3446	gabapentine	Sigma	C1CCC(CC1)(CC(=O)O)CN	88.7	87.0
81	3469	gentisic acid	Sigma	C1=CC(=C(C=C1O)C(=O)O)O	98.4	93.2
82	3559	haloperidol	Sigma	C1CN(CCC1(C2=CC=C(C=C2)Cl)O)CCCC(=O)C3=CC=C(C=C3)F	161.0	339.8
83	3658	hydroxyzine	VWR	C1CN(CCN1CCOCCO)C(C2=CC=CC=C2)C3=CC=C(C=C3)Cl	102.5	113.4
84	3672	ibuprofen	Sigma	CC(C)CC1=CC=C(C=C1)C(C)C(=O)O	97.8	95.1
85	3676	lidocaine	Sigma	CCN(CC)CC(=O)NC1=C(C=CC=C1C)C	92.7	98.6
86	3678	alverine	Sigma	CCN(CCCC1=CC=CC=C1)CCCC2=CC=CC=C2	107.1	118.2
87	3696	imipramine	Sigma	CN(C)CCCN1C2=CC=CC=C2CCC3=CC=CC=C31	115.5	253.6
88	3715	indomethacin	Sigma	CC1=C(C2=C(N1C(=O)C3=CC=C(C=C3)Cl)C=CC(=C2)OC)CC(=O)O	113.6	103.3
89	3783	isoxsuprine	Sigma	CC(COC1=CC=CC=C1)NC(C)C(C2=CC=C(C=C2)O)O	101.0	96.5
90	3827	ketotifene	Sigma	CN1CCC(=C2C3=C(C(=O)CC4=CC=CC=C42)SC=C3)CC1	101.5	136.8
91	3869	labetalol	Sigma	CC(CCC1=CC=CC=C1)NCC(C2=CC(=C(C=C2)O)C(=O)N)O	113.5	134.4
92	3878	lamotrigine	Sigma	C1=CC(=C(C(=C1)Cl)Cl)C2=C(N=C(N=N2)N)N	117.1	100.1
93	3947	lofepramine	Sigma	CN(CCCN1C2=CC=CC=C2CCC3=CC=CC=C31)CC(=O)C4=CC=C(C=C4)Cl	143.9	148.2
94	3955	loperamide	Sigma	CN(C)C(=O)C(CCN1CCC(CC1)(C2=CC=C(C=C2)Cl)O)(C3=CC=CC=C3)C4=CC=CC=C4	490.5	441.0
95	3957	loratadine	Sigma	CCOC(=O)N1CCC(=C2C3=C(CCC4=C2N=CC=C4)C=C(C=C3)Cl)CC1	121.5	120.4
96	4011	maprotiline	Sigma	CNCCCC12CCC(C3=CC=CC=C31)C4=CC=CC=C24	164.5	341.7
97	4031	mebeverine	Sigma	CCN(CCCCOC(=O)C1=CC(=C(C=C1)OC)OC)C(C)CC2=CC=C(C=C2)OC	120.7	147.3
98	4032	mecamylamine	Sigma	CC1(C2CCC(C2)C1(C)NC)C	106.4	89.8
99	4037	parkemed	Sigma	CC1=C(C(=C(C=C1)Cl)NC2=CC=CC=C2C(=O)O)Cl	80.7	93.8
100	4044	meclofenamate	Sigma	CC1=C(C(=CC=C1)NC2=CC=CC=C2C(=O)O)C	103.2	87.2
101	4054	memantine	Sigma	CC12CC3CC(C1)(CC(C3)(C2)N)C	97.1	110.2
102	4078	mesoridazine	Sigma	CN1CCCCC1CCN2C3=CC=CC=C3SC4=C2C=C(C=C4)S(=O)C	134.6	125.2

103	4098	methapyrilene	Sigma	CN(C)CCN(CC1=CC=CS1)C2=CC=CC=N2	88.9	111.4
104	4139	methene blue	Sigma	CN(C)C1=CC2=C(C=C1)N=C3C=CC(=[N+](C)C)C=C3S2	83.1	60.3
105	4168	metoclopramide	Sigma	CCN(CC)CCNC(=O)C1=CC(=C(C=C1OC)N)Cl	113.2	92.8
106	4184	mianserine	Sigma	CN1CCN2C(C1)C3=CC=CC=C3CC4=CC=CC=C42	98.8	101.4
107	4205	mirtazapine	Sigma	CN1CCN2C(C1)C3=CC=CC=C3CC4=C2N=CC=C4	95.5	103.2
108	4211	mitotane	Sigma	C1=CC=C(C(=C1)C(C2=CC=C(C=C2)Cl)C(Cl)Cl)Cl	107.5	112.4
109	4260	moxisylyte	Sigma	CC1=CC(=C(C=C1OC(=O)C)C(C)C)OCCN(C)C	107.2	87.8
110	4375	fg-7142	Sigma	CNC(=O)C1=NC=C2C(=C1)C3=CC=CC=C3N2	90.0	102.8
111	4436	naphazoline	Sigma	C1CN=C(N1)CC2=CC=CC3=CC=CC=C32	94.4	91.2
112	4541	norfluoxetine	Sigma	C1=CC=C(C=C1)C(CCN)OC2=CC=C(C=C2)C(F)(F)F	182.8	583.3
113	4543	nortriptyline	Sigma	CNCCC=C1C2=CC=CC=C2CCC3=CC=CC=C31	191.7	814.1
114	4544	noscapine	Sigma	CN1CCC2=CC3=C(C(=C2C1C4C5=C(C(=C(C=C5)OC)OC)C(=O)O4)OC)OCO3	92.8	89.4
115	4547	repaglinide	Sigma	CCOC1=C(C=CC(=C1)CC(=O)NC(CC(C)C)C2=CC=CC=C2N3CCCCC3)C(=O)O	102.8	105.1
116	4594	omeprazole	Sigma	CC1=CN=C(C(=C1OC)C)CS(=O)C2=NC3=C(N2)C=C(C=C3)OC	97.3	83.2
117	4601	orphenadrine	Sigma	CC1=CC=CC=C1C(C2=CC=CC=C2)OCCN(C)C	116.5	121.4
118	4619	oxeladin	Sigma	CCC(CC)(C1=CC=CC=C1)C(=O)OCCOCCN(CC)CC	131.7	145.4
119	4624	6-hydroxydopamine	Sigma	C1=C(C(=CC(=C1O)O)O)CCN	116.6	108.5
120	4634	oxybutynine	Sigma	CCN(CC)CC#CCOC(=O)C(C1CCCCC1)(C2=CC=CC=C2)O	122.5	140.4
121	4636	oxymetazoline	Sigma	CC1=CC(=C(C(=C1CC2=NCCN2)C)O)C(C)(C)C	115.7	116.6
122	4642	daricon	Sigma	CN1CCCN=C1COC(=O)C(C2CCCCC2)(C3=CC=CC=C3)O	104.1	108.2
123	4679	pantoprazole	Sigma	COC1=C(C(=NC=C1)CS(=O)C2=NC3=C(N2)C=C(C=C3)OC(F)F)OC	111.6	111.2
124	4680	papaverine	Sigma	COC1=C(C=C(C=C1)CC2=NC=CC3=CC(=C(C=C32)OC)OC)OC	90.8	93.5
125	4687	1,7-dimethylxanthine	Sigma	CN1C=NC2=C1C(=O)N(C(=O)N2)C	104.6	88.9
126	4746	perhexiline	Sigma	C1CCC(CC1)C(CC2CCCCN2)C3CCCCC3	255.6	411.2
127	4748	perphenazine	Sigma	C1CN(CCN1CCCN2C3=CC=CC=C3SC4=C2C=C(C=C4)Cl)CCO	115.4	201.0
128	4754	phenacetin	Sigma	CCOC1=CC=C(C=C1)NC(=O)C	93.3	103.7
129	4767	phenothrin	Sigma	CC(=CC1C(C1(C)C)C(=O)OCC2=CC(=CC=C2)OC3=CC=CC=C3)C	104.8	109.5
130	4784	phenylmethylsulfonylfluoride	Calbiochem	C1=CC=C(C=C1)CS(=O)(=O)F	100.0	104.5
131	4830	pipamperone	Sigma	C1CCN(CC1)C2(CCN(CC2)CCCC(=O)C3=CC=C(C=C3)F)C(=O)N	103.9	90.6
132	4847	pirenperone	Sigma	CC1=C(C(=O)N2C=CC=CC2=N1)CCN3CCC(CC3)C(=O)C4=CC=C(C=C4)F	88.7	100.4
133	4904	pridinole	Sigma	C1CCN(CC1)CCC(C2=CC=CC=C2)(C3=CC=CC=C3)O	132.7	104.6
134	4917	compazine	Sigma	CN1CCN(CC1)CCCN2C3=CC=CC=C3SC4=C2C=C(C=C4)Cl	151.6	235.2
135	4919	procyclidine	Sigma	C1CCC(CC1)C(CCN2CCCC2)(C3=CC=CC=C3)O	104.2	97.4
136	4920	progesterone	Sigma	CC(=O)C1CCC2C1(CCC3C2CCC4=CC(=O)CCC34C)C	97.2	95.2
137	4926	promazine	Sigma	CN(C)CCCN1C2=CC=CC=C2SC3=CC=CC=C31	101.5	136.8
138	4927	promethazine	Sigma	CC(CN1C2=CC=CC=C2SC3=CC=CC=C31)N(C)C	91.1	234.7
139	4932	propafenone	Sigma	CCCNCC(COC1=CC=CC=C1C(=O)CCC2=CC=CC=C2)O	116.1	97.8
140	4935	proparacaine	Sigma	CCCOC1=C(C=C(C=C1)C(=O)OCCN(CC)CC)N	98.1	114.4
141	4946	propranolol	Sigma	CC(C)NCC(COC1=CC=CC2=CC=CC=C21)O	28.9	125.1
142	4971	protoporphyrine IX	Sigma	CC1=C(C2=CC3=C(C(=C(N3)C=C4C(=C(C(=N4)C=C5C(=C(C(=N5)C=C1N2)C)CCC(=O)O)CCC(=O)O)C)C=C)C)C=C	61.4	47.4
143	4976	protriptyline	Sigma	CNCCCC1C2=CC=CC=C2C=CC3=CC=CC=C13	108.8	141.3
144	4992	pyrilamine	Sigma	CN(C)CCN(CC1=CC=C(C=C1)OC)C2=CC=CC=N2	100.7	107.4
145	5002	quetiapine	AstraZenca	C1CN(CCN1CCOCCO)C2=NC3=CC=CC=C3SC4=CC=CC=C42	79.1	105.3
146	5035	raloxifene	Tocris	C1CCN(CC1)CCOC2=CC=C(C=C2)C(=O)C3=C(SC4=C3C=CC(=C4)O)C5=CC=C(C=C5)O	265.4	224.8
147	5071	flumadine	Sigma	CC(C12CC3CC(C1)CC(C3)C2)N	112.3	116.7
148	5074	ritanserine	Sigma	CC1=C(C(=O)N2C=CSC2=N1)CCN3CCC(=C(C4=CC=C(C=C4)F)C5=CC=C(C=C5)F)CC3	97.5	92.6
149	5092	rolipram	Biotrend	COC1=C(C=C(C=C1)C2CC(=O)NC2)OC3CCCC3	106.6	98.3
150	5095	ropinirole	VWR	CCCN(CCC)CCC1=C2CC(=O)NC2=CC=C1	101.8	107.9
151	5142	S-methylisothiourea	Calbiochem	CSC(=N)N	111.1	100.7
152	5152	salmeterole	Sigma	C1=CC=C(C=C1)CCCCOCCCCCCNCC(C2=CC(=C(C=C2)O)CO)O	106.6	188.3
153	5265	spiperone	Sigma	C1CN(CCC12C(=O)NCN2C3=CC=CC=C3)CCCC(=O)C4=CC=C(C=C4)F	150.7	91.8

154	5354	suloctidil	Sigma	CCCCCCCCNC(C)C(C1=CC=C(C=C1)SC(C)C)O	547.9	591.1
155	5355	sulpiride	Sigma	CCN1CCCC1CNC(=O)C2=C(C=CC(=C2)S(=O)(=O)N)OC	100.4	102.1
156	5361	suramine	Sigma	CC1=C(C=C(C=C1)C(=O)NC2=C3C(=CC(=CC3=C(C=C2)S(=O)(=O)O)S(=O)(=O)O)S(=O)(=O)O)NC(=O)C4=CC(=CC=C4)NC(=O)NC5=CC=CC(=C5)C(=O)NC6=C(C=CC(=C6)C(=O)NC7=C8C(=CC(=CC8=C(C=C7)S(=O)(=O)O)S(=O)(=O)O)S(=O)(=O)O)C	104.8	104.4
157	5411	tetracaine	Sigma	CCCCNC1=CC=C(C=C1)C(=O)OCCN(C)C	108.9	101.9
158	5452	thioridazine	Sigma	CN1CCCCC1CCN2C3=CC=CC=C3SC4=C2C=C(C=C4)SC	166.7	328.4
159	5475	tilorone	Sigma	CCN(CC)CCOC1=CC2=C(C=C1)C3=C(C2=O)C=C(C=C3)OCCN(CC)CC	499.3	616.9
160	5496	tobramycine	Sigma	C1C(C(C(C(C1N)OC2C(C(C(C(O2)CO)O)N)O)O)OC3C(CC(C(O3)CN)O)N)N	99.6	100.1
161	5502	tofisopam	Sigma	CCC1C(=NN=C(C2=CC(=C(C=C12)OC)OC)C3=CC(=C(C=C3)OC)OC)C	108.3	105.0
162	5514	topiramate	Sigma	CC1(OC2COC3(C(C2O1)OC(O3)(C)C)COS(=O)(=O)N)C	81.3	68.7
163	5566	trifluoperazine	Sigma	CN1CCN(CC1)CCCN2C3=CC=CC=C3SC4=C2C=C(C=C4)C(F)(F)F	495.5	430.3
164	5568	triflupromazine	Sigma	CN(C)CCCN1C2=CC=CC=C2SC3=C1C=C(C=C3)C(F)(F)F	130.8	430.9
165	5572	trihexyphenidyl	Sigma	C1CCC(CC1)C(CCN2CCCCC2)(C3=CC=CC=C3)O	117.4	108.5
166	5584	trimipramine	Sigma	CC(CN1C2=CC=CC=C2CCC3=CC=CC=C31)CN(C)C	126.4	249.9
167	5587	tripelennamine	Sigma	CN(C)CCN(CC1=CC=CC=C1)C2=CC=CC=N2	98.1	109.7
168	5606	tulobuterol	Sigma	CC(C)(C)NCC(C1=CC=CC=C1Cl)O	108.3	103.7
169	5656	venlafaxine	Sigma	CN(C)CC(C1=CC=C(C=C1)OC)C2(CCCCC2)O	100.3	112.6
170	5668	vincamine	Sigma	CCC12CCCN3C1C4=C(CC3)C5=CC=CC=C5N4C(C2)(C(=O)OC)O	106.3	111.8
171	5681	N-(6-aminohexyl)-5-chloro-1-naphthalene	Sigma	C1=CC2=C(C=CC=C2Cl)C(=C1)S(=O)(=O)NCCCCCCN	101.7	102.4
172	5709	xylomtazoline	Sigma	CC1=CC(=CC(=C1CC2=NCCN2)C)C(C)(C)C	118.7	119.4
173	5717	zafirlukast	VWR	CC1=CC=CC=C1S(=O)(=O)NC(=O)C2=CC(=C(C=C2)CC3=CN(C4=C3C=C(C=C4)NC(=O)OC5CCCC5)C)OC	103.3	96.0
174	5743	dexamethasone	Sigma	CC1CC2C3CCC4=CC(=O)C=CC4(C3(C(CC2(C1(C(=O)CO)O)C)O)F)C	85.3	85.0
175	5753	corticosterone	Sigma	CC12CCC(=O)C=C1CCC3C2C(CC4(C3CCC4C(=O)CO)C)O	90.3	110.0
176	5754	hydrochortisone	Sigma	CC12CCC(=O)C=C1CCC3C2C(CC4(C3CCC4(C(=O)CO)O)C)O	81.1	91.4
177	5775	phentolamine	Sigma	CC1=CC=C(C=C1)N(CC2=NCCN2)C3=CC(=CC=C3)O	96.3	97.0
178	5793	glucose	Sigma	C(C1C(C(C(C(O1)O)O)O)O)O	95.8	104.9
179	5857	lynestrenole	Biotrend	CC12CCC3C(C1CCC2(C#C)O)CCC4=CCCCC34	101.7	102.1
180	5902	camylofine	Sigma	CCN(CC)CCNC(C1=CC=CC=C1)C(=O)OCCC(C)C	123.9	267.8
181	5959	chloramphenicole	Sigma	C1=CC(=CC=C1C(C(CO)NC(=O)C(Cl)Cl)O)[N+](=O)[O-]	89.3	90.3
182	5978	vincristine	Sigma	CCC1(CC2CC(C3=C(CCN(C2)C1)C4=CC=CC=C4N3)(C5=C(C=C6C(=C5)C78CCN9C7C(C=CC9)(C(C8N6C=O)(C(=O)OC)O)OC(=O)C)CC)OC)C(=O)OC)O	111.9	27.2
183	6029	uridine	Sigma	C1=CN(C(=O)NC1=O)C2C(C(C(O2)CO)O)O	115.9	101.8
184	6167	colchicine	Sigma	CC(=O)NC1CCC2=CC(=C(C(=C2C3=CC=C(C(=O)C=C13)OC)OC)OC)OC	39.7	38.1
185	6251	D-mannitol	Sigma	C(C(C(C(C(CO)O)O)O)O)O	95.9	106.0
186	6301	chlorquinaldol	Chemos	CC1=NC2=C(C=C1)C(=CC(=C2O)Cl)Cl	101.2	81.9
187	6536	triparanol	Sigma	CCN(CC)CCOC1=CC=C(C=C1)C(CC2=CC=C(C=C2)Cl)(C3=CC=C(C=C3)C)O	432.8	683.3
188	6691	warfarine	Sigma	CC(=O)CC(C1=CC=CC=C1)C2=C(OC3=CC=CC=C3C2=O)O	97.6	97.6
189	6729	buclizine	VWR	CC(C)(C)C1=CC=C(C=C1)CN2CCN(CC2)C(C3=CC=CC=C3)C4=CC=C(C=C4)Cl	109.5	106.4
190	6832	benztropine	VWR	CN1C2CCC1CC(C2)OC(C3=CC=CC=C3)C4=CC=CC=C4	122.1	143.8
191	6834	brompheniramine	Sigma	CN(C)CCC(C1=CC=C(C=C1)Br)C2=CC=CC=N2	107.3	97.0
192	8233	erythromycine	Sigma	CCC1C(C(C(C(=O)C(CC(C(C(C(C(C(=O)O1)C)OC2CC(C(C(O2)C)O)(C)OC)C)OC3C(C(CC(O3)C)N(C)C)O)(C)O)C)C)O)(C)O	97.0	110.7
193	8549	quinine	Sigma	COC1=CC2=C(C=CN=C2C=C1)C(C3CC4CCN3CC4C=C)O	110.7	112.5
194	8969	yohimbine	Sigma	COC(=O)C1C(CCC2C1CC3C4=C(CCN3C2)C5=CC=CC=C5N4)O	115.1	80.5
195	9417	opipramol	Sigma	C1CN(CCN1CCCN2C3=CC=CC=C3C=CC4=CC=CC=C42)CCO	117.8	137.6
196	9705	ay-9944	Sigma	C1CC(CCC1CNCC2=CC=CC=C2Cl)CNCC3=CC=CC=C3Cl	406.6	575.6
197	10114	enoxolone	Sigma	CC1(C2CCC3(C(C2(CCC1O)C)C(=O)C=C4C3(CCC5(C4CC(CC5)(C)C(=O)O)C)C)C)C	108.0	99.8
198	10219	emetine	VWR	CCC1CN2CCC3=CC(=C(C=C3C2CC1CC4C5=CC(=C(C=C5CCN4)OC)OC)OC)OC	60.6	66.6
199	11289	chlorotrianisene	Sigma	COC1=CC=C(C=C1)C(=C(C2=CC=C(C=C2)OC)Cl)C3=CC=C(C=C3)OC	116.9	117.1
200	13738	oxolamine	Sigma	CCN(CC)CCC1=NC(=NO1)C2=CC=CC=C2	92.6	94.8

201	15443	azaperone	Sigma	C1CN(CCN1CCCC(=O)C2=CC=C(C=C2)F)C3=CC=CC=N3	101.7	91.4
202	16362	pimozide	Sigma	C1CN(CCC1N2C3=CC=CC=C3NC2=O)CCCC(C4=CC=C(C=C4)F)C5=CC=C(C=C5)F	322.5	574.7
203	19143	cyclazocine	Sigma	CC1C2CC3=C(C1(CCN2CC4CC4)C)C=C(C=C3)O	88.0	89.4
204	22530	mebhydroline	Sigma	CN1CCC2=C(C1)C3=CC=CC=C3N2CC4=CC=CC=C4	107.8	139.1
205	25249	stanazolol	Sigma	CC12CCC3C(C1CCC2(C)O)CCC4C3(CC5=C(C4)NN=C5)C	103.4	92.3
206	25295	chloropyramine	Sigma	CN(C)CCN(CC1=CC=C(C=C1)Cl)C2=CC=CC=N2	103.9	106.0
207	26757	selegeline	Sigma	CC(CC1=CC=CC=C1)N(C)CC#C	104.6	91.7
208	26987	clemastine	Chemos	CC(C1=CC=CC=C1)(C2=CC=C(C=C2)Cl)OCCC3CCCN3C	214.0	255.8
209	28486	lithium	Sigma	[Li+]	97.2	98.0
210	30323	danorubicine	Sigma	CC1C(C(CC(O1)OC2CC(CC3=C(C4=C(C(=C23)O)C(=O)C5=C(C4=O)C=CC=C5OC)O)(C(=O)C)O)N)O	31.0	112.6
211	31101	bromocriptine	Sigma	CC(C)CC1C(=O)N2CCCC2C3(N1C(=O)C(O3)(C(C)C)NC(=O)C4CN(C5CC6=C(NC7=CC=CC(=C67)C5=C4)Br)C)O	110.5	95.9
212	33625	indoramine	Sigma	C1CN(CCC1NC(=O)C2=CC=CC=C2)CCC3=CNC4=CC=CC=C43	124.2	68.4
213	33630	penfluridol	Sigma	C1CN(CCC1(C2=CC(=C(C=C2)Cl)C(F)(F)F)O)CCCC(C3=CC=C(C=C3)F)C4=CC=C(C=C4)F	381.0	536.3
214	33741	tramadol	Sigma	CN(C)CC1CCCCC1(C2=CC(=CC=C2)OC)O	100.8	85.8
215	36339	etomidate	Sigma	CCOC(=O)C1=CN=CN1C(C)C2=CC=CC=C2	107.3	97.5
216	39186	diltiazem	Sigma	CC(=O)OC1C(SC2=CC=CC=C2N(C1=O)CCN(C)C)C3=CC=C(C=C3)OC	103.6	101.3
217	40973	desogstrel	Sigma	CCC12CC(=C)C3C(C1CCC2(C#C)O)CCC4=CCCCC34	106.0	93.8
218	43815	paroxetine	Sigma	C1CNCC(C1C2=CC=C(C=C2)F)COC3=CC4=C(C=C3)OCO4	160.2	208.0
219	47811	peroglide	Sigma	CCCN1CC(CC2C1CC3=CNC4=CC=CC2=C34)CSC	70.1	99.5
220	48041	encainide	VWR	CN1CCCCC1CCC2=CC=CC=C2NC(=O)C3=CC=C(C=C3)OC	97.7	101.3
221	54385	levocabastine	Sigma	CC1CN(CCC1(C2=CC=CC=C2)C(=O)O)C3CCC(CC3)(C#N)C4=CC=C(C=C4)F	123.3	92.6
222	55245	mifepristone	Sigma	CC#CC1(CCC2C1(CC(C3=C4CCC(=O)C=C4CCC23)C5=CC=C(C=C5)N(C)C)C)O	113.2	113.6
223	55891	fosinopril	VWR	CCC(=O)OC(C(C)C)OP(=O)(CCCCC1=CC=CC=C1)CC(=O)N2CC(CC2C(=O)O)C3CCCCC3	100.5	83.9
224	57469	imquimod	Sigma	CC(C)CN1C=NC2=C1C3=CC=CC=C3N=C2N	110.0	121.1
225	60149	sertindole	Sigma	C1CN(CCC1C2=CN(C3=C2C=C(C=C3)Cl)C4=CC=C(C=C4)F)CCN5CCNC5=O	346.4	829.4
226	60648	tiagabine	VWR	CC1=C(SC=C1)C(=CCCN2CCCC(C2)C(=O)O)C3=C(C=CS3)C	104.6	96.4
227	60663	mibefradil	Sigma	CC(C)C1C2=C(CCC1(CCN(C)CCCC3=NC4=CC=CC=C4N3)OC(=O)COC)C=C(C=C2)F	419.3	553.1
228	60854	ziprasidone	Sigma	C1CN(CCN1CCC2=C(C=C3C(=C2)CC(=O)N3)Cl)C4=NSC5=CC=CC=C54	126.4	129.2
229	64143	nelfinavir	Chemos	CC1=C(C=CC=C1O)C(=O)NC(CSC2=CC=CC=C2)C(CN3CC4CCCCC4CC3C(=O)NC(C)(C)C)O	101.3	96.2
230	64757	zimelidine	Sigma	CN(C)CC=C(C1=CC=C(C=C1)Br)C2=CN=CC=C2.Cl.Cl	106.2	104.9
231	65105	L-leucinemethylester	Sigma	CC(C)CC(C(=O)OC)N	89.2	113.9
232	65576	tomatidine	Sigma	CC1CCC2(C(C3C(O2)CC4C3(CCC5C4CCC6C5(CCC(C6)O)C)C)C)NC1	397.2	379.6
233	65856	reboxetine	Sigma	CCOC1=CC=CC=C1OC(C2CNCCO2)C3=CC=CC=C3	103.7	101.1
234	65866	lercanidipine	Sigma	CC1=C(C(C(=C(N1)C)C(=O)OC(C)(C)CN(C)CCC(C2=CC=CC=C2)C3=CC=CC=C3)C4=CC(=CC=C4)[N+](=O)[O-])C(=O)OC	121.8	195.7
235	65884	barnidipine	Chemos	CC1=C(C(C(=C(N1)C)C(=O)OC2CCN(C2)CC3=CC=CC=C3)C4=CC(=CC=C4)[N+](=O)[O-])C(=O)OC	145.2	176.0
236	65999	telmisartane	Sigma	CCCC1=NC2=C(C=C(C=C2N1CC3=CC=C(C=C3)C4=CC=CC=C4C(=O)O)C5=NC6=CC=CC=C6N5C)C	124.2	125.0
237	67425	gallamine	VWR	CCN(CC)CCOC1=C(C(=CC=C1)OCCN(CC)CC)OCCN(CC)CC	169.1	105.3
238	68617	sertraline	Tocris	CNC1CCC(C2=CC=CC=C12)C3=CC(=C(C=C3)Cl)Cl	484.8	723.3
239	68876	cloricromene	Sigma	CCN(CC)CCC1=C(C2=C(C(=C(C=C2)OCC(=O)OCC)Cl)OC1=O)C	108.6	95.9
240	70029	dibenzosuberane	Sigma	C1CC2=CC=CC=C2CC3=CC=CC=C31	94.2	99.8
241	74989	atovaquone	Chemos	C1CC(CCC1C2=CC=C(C=C2)Cl)C3=C(C4=CC=CC=C4C(=O)C3=O)O	95.8	94.3
242	74990	topotecine	Sigma	CCC1=C2C=C(C=CC2=NC3=C1CN4C3=CC5=C(C4=O)COC(=O)C5(CC)O)OC(=O)N6CCC(CC6)N7CCCCC7.Cl	85.7	48.6
243	84029	clarithromycine	Sigma	CCC1C(C(C(C(=O)C(CC(C(C(C(C(C(=O)O1)C)OC2CC(C(C(O2)C)O)(C)OC)C)OC3C(C(CC(O3)C)N(C)C)O)(C)OC)C)C)O)(C)O	124.5	112.9
244	91270	moexipril	Sigma	CCOC(=O)C(CCC1=CC=CC=C1)NC(C)C(=O)N2CC3=CC(=C(C=C3CC2C(=O)O)OC)OC	116.0	101.9
245	91769	zolantidine	Tocris	C1CCN(CC1)CC2=CC(=CC=C2)OCCCNC3=NC4=CC=CC=C4S3	120.0	155.0
246	104741	fluvestrant	Sigma	CC12CCC3C(C1CCC2O)C(CC4=C3C=CC(=C4)O)CCCCCCCCCS(=O)CCCC(C(F)(F)F)(F)F	135.2	144.1
247	115100	pranlukast	Tocris	C1=CC=C(C=C1)CCCCOC2=CC=C(C=C2)C(=O)NC3=CC4=C(C=C3)C(=O)C=C(O4)C5=NNN=N5	104.4	116.5

248	124087	desloratadine	Sigma	C1CC2=C(C=CC(=C2)Cl)C(=C3CCNCC3)C4=C1C=CC=N4	208.4	261.3
249	152945	dutasteride	Chemos	CC12CCC3C(C1CCC2C(=O)NC4=C(C=CC(=C4)C(F)(F)F)C(F)(F)F)CCC5C3(C=CC(=O)N5)C	99.6	106.6
250	156391	naproxen	Sigma	CC(C1=CC2=C(C=C1)C=C(C=C2)OC)C(=O)O	113.3	88.3
251	174174	atropine	Sigma	CN1C2CCC1CC(C2)OC(=O)C(CO)C3=CC=CC=C3	98.5	83.5
252	192706	phenserine	Tocris	CC12CCN(C1N(C3=C2C=C(C=C3)OC(=O)NC4=CC=CC=C4)C)C	112.5	101.8
253	197033	dimebone	Aurora Feinchemie	CC1=CC2=C(C=C1)N(C3=C2CN(CC3)C)CCC4=CN=C(C=C4)C	96.5	101.0
254	235905	allylestrenol	Chemos	CC12CCC3C(C1CCC2(CC=C)O)CCC4=CCCCC34	101.6	93.6
255	241903	vinblastine	Chemos	CCC1(CC2CC(C3=C(CCN(C2)C1)C4=CC=CC=C4N3)(C5=C(C=C6C(=C5)C78CCN9C7C(C=CC9)(C(C(C8N6C)(C(=O)OC)O)OC(=O)C)CC)OC)C(=O)OC)O	107.5	126.4
256	360849	cepharanthine	Chemos	CN1CCC2=CC3=C(C4=C2C1CC5=CC=C(C=C5)OC6=C(C=CC(=C6)CC7C8=CC(=C(C=C8CCN7C)OC) O4)OC)OCO3	851.3	565.1
257	424898	propidium	Molecular Probes	CC[N+](C)(CC)CCCN1C(C2=C(C=CC(=C2)N)C3=C1C=C(C=C3)N)C4=CC=CC=C4	90.3	104.9
258	441082	connesine	Sigma	CC1C2CCC3C2(CCC4C3CC=C5C4(CCC(C5)N(C)C)C)CN1C	290.9	610.5
259	442985	solasodine	Chemos	CC1CCC2(C(C3C(O2)CC4C3(CCC5C4CC=C6C5(CCC(C6)O)C)C)C)NC1	108.1	87.1
260	443955	vinpocetine	Sigma	CCC12CCCN3C1C4=C(CC3)C5=CC=CC=C5N4C(=C2)C(=O)OCC	108.5	108.9
261	444008	tibolone	Sigma	CC1CC2=C(CCC(=O)C2)C3C1C4CCC(C4(CC3)C)(C#C)O	109.1	98.8
262	445354	retinol	Sigma	CC1=C(C(CCC1)(C)C)C=CC(=CC=CC(=CCO)C)C	108.5	102.0
263	447043	azithromycine	Sigma	CCC1C(C(C(N(CC(CC(C(C(C(C(C(=O)O1)C)OC2CC(C(C(O2)C)O)(C)OC)C)OC3C(C(CC(O3)C)N(C)C) O)(C)O)C)C)C)O)(C)O	166.3	213.2
264	456201	ketoconazole	Sigma	CC(=O)N1CCN(CC1)C2=CC=C(C=C2)OCC3COC(O3)(CN4C=CN=C4)C5=C(C=C(C=C5)Cl)Cl	117.0	110.1
265	636397	pirarubicin	Sigma	CC1C(C(CC(O1)OC2CC(CC3=C(C4=C(C(=C23)O)C(=O)C5=C(C4=O)C=CC=C5OC)O)(C(=O)CO)O)N) OC6CCCCO6	71.0	79.0
266	636403	carbenoxolone	Sigma	CC1(C2CCC3(C(C2(CCC1OC(=O)CCC(=O)O)C)C(=O)C=C4C3(CCC5(C4CC(CC5)(C)C(=O)O)C)C)C)C	116.1	90.6
267	644020	sparteine	Sigma	C1CCN2CC3CC(C2C1)CN4C3CCCC4	88.7	95.0
268	656586	cerocral	Sigma	CC(C(C1=CC=C(C=C1)O)O)N2CCC(CC2)CC3=CC=CC=C3.CC(C(C1=CC=C(C=C1)O)O)N2CCC(CC2) CC3=CC=CC=C3.C(C(C(=O)O)O)(C(=O)O)O	126.5	130.9
269	667467	chlorprothixene	Sigma	CN(C)CCC=C1C2=CC=CC=C2SC3=C1C=C(C=C3)Cl	126.8	229.4
270	667476	dienesterol	Chemos	CC=C(C1=CC=C(C=C1)O)C(=CC)C2=CC=C(C=C2)O	89.0	105.4
271	667477	doxepin	Sigma	CN(C)CCC=C1C2=CC=CC=C2COC3=CC=CC=C31	93.8	120.4
272	688585	triprolidine	Sigma	CC1=CC=C(C=C1)C(=CCN2CCCC2)C3=CC=CC=N3	102.7	101.1
273	941361	flunarizine	Sigma	C1CN(CCN1CC=CC2=CC=CC=C2)C(C3=CC=C(C=C3)F)C4=CC=C(C=C4)F	141.4	142.2
274	1547484	cinnarizine	Sigma	C1CN(CCN1CC=CC2=CC=CC=C2)C(C3=CC=CC=C3)C4=CC=CC=C4	111.8	123.0
275	1548887	sulindac	Sigma	CC1=C(C2=C(C1=CC3=CC=C(C=C3)S(=O)C)C=CC(=C2)F)CC(=O)O	93.8	107.3
276	1548955	clomiphene	Sigma	CCN(CC)CCOC1=CC=C(C=C1)C(=C(C2=CC=CC=C2)Cl)C3=CC=CC=C3	832.6	894.2
277	2733526	tamoxifen	Sigma	CCC(=C(C1=CC=CC=C1)C2=CC=C(C=C2)OCCN(C)C)C3=CC=CC=C3	740.5	900.1
278	3000226	fusidic acid	Sigma	CC1C2CCC3(C(C2(CCC1O)C)C(CC4C3(CC(C4=C(CCC=C(C)C)C(=O)O)OC(=O)C)C)O)C	114.7	101.4
279	3001386	thiocarlide	VWR	CC(C)CCOC1=CC=C(C=C1)NC(=S)NC2=CC=C(C=C2)OCCC(C)C	102.1	95.3
280	3946663	sb-222200	Sigma	CCC(C1=CC=CC=C1)NC(=O)C2=C(C(=NC3=CC=CC=C32)C4=CC=CC=C4)C	102.7	114.8
281	5280953	harmine	Sigma	CC1=NC=CC2=C1NC3=C2C=CC(=C3)OC	107.3	98.6
282	5281040	montelukast	Biotrend	CC(C)(C1=CC=CC=C1CCC(C2=CC=CC(=C2)C=CC3=NC4=C(C=CC(=C4)Cl)C=C3)SCC5(CC5)CC(=O) O)O	94.2	96.7
283	5281613	diosmine	Sigma	CC1C(C(C(C(O1)OCC2C(C(C(C(O2)OC3=CC(=C4C(=C3)OC(=CC4=O)C5=CC(=C(C=C5)OC)O)O)O)O) O)O)O)O	108.5	103.4
284	5281881	flupenthixol	Sigma	C1CN(CCN1CCC=C2C3=CC=CC=C3SC4=C2C=C(C=C4)C(F)(F)F)CCO	344.5	335.8
285	5282138	cilnidipine	Sigma	CC1=C(C(C(=C(N1)C)C(=O)OCC=CC2=CC=CC=C2)C3=CC(=CC=C3)[N+](=O)[O-])C(=O)OCCOC	121.8	147.9
286	5284514	acrivastine	Biotrend	CC1=CC=C(C=C1)C(=CCN2CCCC2)C3=CC=CC(=N3)C=CC(=O)O	105.6	91.8
287	5288783	calcipotriol	Sigma	CC(C=CC(C1CC1)O)C2CCC3C2(CCCC3=CC=C4CC(CC(C4=C)O)O)C	121.6	122.3
288	5318127	hydrochinine	Sigma	CCC1CN2CCC1CC2C(C3=C4C=C(C=CC4=NC=C3)OC)O	115.6	130.9
289	5324346	fluvoxamine	Sigma	COCCCCC(=NOCCN)C1=CC=C(C=C1)C(F)(F)F	138.1	232.3
290	5360696	dextromethorphan	VWR	CN1CCC23CCCCC2C1CC4=C3C=C(C=C4)OC	105.7	118.4
291	5361092	butorphanol	Sigma	C1CCC2(C3CC4=C(C2(C1)CCN3CC5CCC5)C=C(C=C4)O)O	111.5	106.1

292	6323490	rifabutine	Sigma	CC1C=CC=C(C(=O)NC2=C3C(=NC4(N3)CCN(CC4)CC(C)C)C5=C6C(=C(C(=C5C2=O)O)C)OC(C6=O)(OC=CC(C(C(C(C(C(C1O)C)O)C)OC(=O)C)C)OC)C)C	130.7	148.8
293	6440717	spiramycine	Sigma	CC1CC=CC=CC(C(CC(C(C(C(CC(=O)O1)O)OC)OC2C(C(C(C(O2)C)OC3CC(C(C(O3)C)O)(C)O)N(C)C)O)CC=O)C)OC4CCC(C(O4)C)N(C)C	107.3	107.2
294	6473883	dirithromycine	Sigma	CCC1C(C2C(C(C(CC(C(C(C(C(C(=O)O1)C)OC3CC(C(C(O3)C)O)(C)OC)C)OC4C(C(CC(O4)C)N(C)C)O)(C)O)C)NC(O2)COCCOC)C)(C)O	99.1	112.0
295	6603696	alaproclate	Sigma	CC(C(=O)OC(C)(C)CC1=CC=C(C=C1)Cl)N	107.7	117.7
296	9552079	chlorhexidine	Sigma	C1=CC(=CC=C1NC(=NC(=NCCCCCCN=C(N)N=C(N)NC2=CC=C(C=C2)Cl)N)N)Cl	101.8	132.5
297	9567573	roxithromycine	Sigma	CCC1C(C(C(C(=NOCOCCOC)C(CC(C(C(C(C(C(=O)O1)C)OC2CC(C(C(O2)C)O)(C)OC)C)OC3C(C(CC(O3)C)N(C)C)O)(C)O)C)C)O)(C)O	116.2	116.1
